# The effect of phenotypic aging on the relationship between cancer history and mortality in US adults

**DOI:** 10.3389/fragi.2025.1710324

**Published:** 2026-01-12

**Authors:** Meng-Hua Tao, Chun-Hui Lin, Shu-Chun Chuang, Wan-Ting Su, Horng-Shiuann Wu

**Affiliations:** 1 Department of Public Health Sciences, Henry Ford Health, Detroit, MI, United States; 2 Department of Epidemiology and Biostatistics, Michigan State University, East Lansing, MI, United States; 3 Institute of Population Health Sciences, National Health Research Institutes, Zhunan, Taiwan; 4 College of Nursing, Michigan State University, East Lansing, MI, United States

**Keywords:** all-cause mortality, biological age, cancer history, cancer-specific mortality, cardiovascular-specific mortality

## Abstract

Cancer survivors may have an accelerated biological aging process compared to cancer-free individuals. In this study, we aimed to investigate associations between clinical measures of biological aging and mortality (all-cause, cancer, and cardiovascular disease [CVD]) and examine whether the association between cancer history and mortality is mediated by biological aging. Data from the National Health and Nutrition Examination Survey (NHANES) 1999–2010, with follow-up through 31 December 2019, were used. A total of 1,493 cancer survivors and 4,479 matched non-cancer individuals aged ≥20 years were included. Cox regression models were used to estimate hazard ratios (HRs) and 95% confidence intervals (CIs). Biological age (BA) was measured by phenotypic age (PhenoAge) based on nine clinical biomarkers. The mediating effect of biological age was assessed using structural equation models with the bootstrapping method by estimating indirect (IE) and direct (DE) effects from cancer history to mortality. Compared to non-cancer individuals, cancer survivors had accelerated PhenoAge. The association between cancer history and all-cause mortality risk was partially mediated by PhenoAge acceleration (HR^IE^ = 1.02, 95% CI: 1.01–1.03). Accelerated PhenoAge also partially mediated the association between cancer history and cancer-specific mortality (HR^IE^ = 1.06, 95% CI: 1.01–1.18). In particular, PhenoAge acceleration mediated 15.5% and 24.1% of the associations of cancer history with all-cause and cancer-specific mortality, respectively. Our results highlight the importance of decelerating the biological aging process among cancer survivors, which may improve survivorship and long-term health in this population.

## Introduction

Cancer risk increases with age, with the median age of cancer diagnosis being 66 (National Cancer Institute, 2021; Zahed et al., 2024). The number of cancer survivors in the United States (US) has increased largely due to advancements in early detection and treatment, with two-thirds (67%) of 18 million American cancer survivors aged ≥65 years (Miller et al., 2022). However, this success has come with the recognition that, compared to individuals without a cancer history, cancer survivors have significantly elevated risks of premature mortality, serious chronic disorders, and early-onset or more frequent occurrence of age-related diseases (Weaver et al., 2012; Lustberg et al., 2023), suggesting an accelerated aging process (Wang et al., 2021; Bhatia et al., 2022; Lustberg et al., 2023). Aging is a complex biological process (Hamczyk et al., 2020). Individuals have heterogeneous rates of aging at the biological level, and the rate of biological aging is an important indicator for predicting long-term health outcomes (Hamczyk et al., 2020). Recent studies have shown that acceleration of biological age (BA), measured by clinical biomarker-based phenotypic age (PhenoAge), is associated with an increased risk of all-cause and cardiovascular disease (CVD)-specific mortality in cancer survivors (Zhang et al., 2022; Wang et al., 2025). Therefore, we hypothesized that cancer history can increase the risk of mortality through its accelerated influence on the biological aging process. To test this hypothesis, data from the National Health and Nutrition Examination Survey (NHANES) were analyzed to investigate associations between biological age and all-cause, CVD-specific, and cancer-specific mortality and examine the role of biological age in the association between cancer history and the risk of mortality.

## Materials and methods

### Study population and outcomes

We utilized data from six consecutive cycles of the NHANES, spanning from 1999 to 2010, to identify cancer survivors among participants aged 20 years and above. The NHANES is an on-going program conducted by the National Center for Health Statistics (NCHS) of the Centers for Disease Control and Prevention (CDC) to access health and nutritional status of a nationally representative sample of the noninstitutionalized civilian resident population of the United States, as described previously in detail (Zipf et al., 2013). In this study, cancer survivors were defined as having cancer if they responded “Yes” to the interview question “Have you ever been told by a doctor or other health professional that you had cancer or a malignancy of any kind?” (n = 2,336). Participants with a non-melanoma or unknown skin cancer were excluded (n = 658), unless they had an additional type of malignancy. Participants were further excluded if they had missing data on mortality follow-up, nine clinical biomarkers, or demographic variables (age, sex, race/ethnicity, and education) (n = 185). Among participants who did not report a cancer diagnostic history and had complete information on mortality follow-up and nine clinical biomarkers, we randomly selected individuals using propensity score matching following a three-to-one (3:1 ratio) nearest neighbor rule by age and sex. Finally, the study cohort consisted of 1,493 adults with a cancer history and 4,479 age- and sex-matched individuals without a cancer history. All participants provided written informed consent, and the Institutional Review Board of the NCHS approved NHANES protocols for all cycles (National Center for Health Statistics, 2024).

Both cancer survivors and matched non-cancer individuals were followed for mortality status from the date of the Mobile Examination Center (MEC) visit until 31 December 2019. Mortality outcomes were determined using matching methods between NHANES and the National Death Index death certificate records described previously (National Center for Health Statistics, 2022). Additionally, the Social Security Administration, Centers for Medicare and Medicaid Services, or death certificate review were used to confirm the mortality status (National Center for Health Statistics, 2022). The 10th revision of the International Statistical Classifications of Disease, Injuries, and Causes of Death (ICD-10) was used to classify deaths from CVD and cancer. All-cause mortality was classified as death from any cause.

### Assessment of biological age

PhenoAge (Levine et al., 2018), a previously developed and validated measure of biological age (BA), was estimated in the current analysis. According to a previously developed method (Levine et al., 2018; Kwon and Belsky, 2021), PhenoAge was estimated based on the parametrization of the Gompertz proportional hazard model on chronological age and the nine biomarkers, namely, albumin, creatinine, glucose, C-reactive protein (CRP), lymphocyte percentage, mean cell volume, red blood cell distribution width, alkaline phosphatase, and white blood cell count, which captured biological functions of inflammation, immunity, or metabolism. To estimate the deviation between PhenoAge and chronological age, we further fitted a linear regression of PhenoAge against chronological age and then computed residual differences between the estimated BA and chronological age as age acceleration. Therefore, a positive or negative value of PhenoAge residual (PhenoAge Acceleration (PhenoAgeAccel)) indicated that a person appears biologically older or younger than her/his chronological age (Levine et al., 2018).

### Statistical analyses

We compared cancer survivors and matched non-cancer individuals based on demographic and health-related characteristics using analysis of the Rao–Scott chi-square test for categorical variables and the *t*-test for continuous variables. Cox proportional hazards regression was applied to estimate hazard ratios (HRs) for associations between PhenoAgeAccel and risk of all-cause, cancer-specific, or CVD-specific mortality. We did not estimate associations between PhenoAgeAccel and the risk of Alzheimer’s disease- or diabetes-specific mortality due to the small number of individuals who died from these diseases. Potential confounders included in multivariable modeling were race/ethnicity; sex (male, female); educational level (high school and less, some college, and college or higher); smoking status (never, former, and current); alcohol drinking status (ever, never); body mass index (BMI, weight (kg)/height (m)^2^); comorbidity index (number of chronic conditions including diabetes, coronary heart disease, cognitive heart failure, heart attack, stroke, hypertension, and liver disease); physical activity level; and daily total energy intake. In NHANES, race/ethnicity was categorized based on survey questions regarding race and Hispanic origin: non-Hispanic White, referring to white people who are not of Hispanic origin; non-Hispanic Black, referring to black people without Hispanic origin; Hispanic, referring to all Hispanic people regardless of race; non-Hispanic Asian, including Asian people without Hispanic origin; and other race, including American Indian or Alaska Native, Native Hawaiian or other Pacific Islander, and multiracial persons. Physical activity was categorized into three strata: inactive, moderate, and active level, defined as no vigorous or moderate physical activity in the past 30 days, at least 10 min of sustained moderate or vigorous physical activity, or at least 10 min of sustained moderate and vigorous physical activity, respectively. The above analyses were repeated in two sex groups (female and male) and in three major types of cancer (breast [female], colon, and prostate [male]). We did not perform analyses among individuals with other types of cancer because of the relatively small sample size (N < 100) with a limited number of outcome events. In sensitivity analyses, cancer survivors who had received a cancer diagnosis ≤5 years prior to entering the study were excluded; analysis was conducted to mitigate the influence of pre-diagnosed biological aging on mortality.

Furthermore, causal mediation analyses were performed to evaluate the potential mediating effect of biological aging (i.e., PhenoAgeAccel) on the association between cancer history and mortality, adjusting for potential confounders, using the R package “regmedint” (Yoshida et al., 2024). Structural equation models with the bootstrapping approach within the counterfactual framework (Valeri and VanderWeele, 2015) were applied. In the model, the total effect (TE) can be decomposed into a direct effect (DE) path from the exposure variable (cancer status) to the outcome variable (i.e., all-cause, CVD-specific, and cancer-specific mortality) and an indirect effect (IE) path from the exposure variable to the mediator (i.e., PhenoAgeAccel) and from the mediator to the outcome. Mediation proportion, the ratio of IE to TE, showed the degree to which the total effect is mediated through the mediator. The total, direct, and indirect effects were estimated on the hazard ratio scale and were conditional on covariates at pre-set levels. We did not perform mediation analyses among individuals with colon, female breast, or prostate cancer because of the relatively small number of outcome events and limited sample size. All statistical tests were two-sided with a significance level of 0.05. R version 4.4.0 (R Foundation for Statistical Computing, Vienna, Austria) and SAS version 9.4 (SAS Institute Inc., Cary, NC) were used to conduct all statistical analyses.

## Results

Among 1,493 cancer survivors (56.3% women) and 4,479 matched non-cancer individuals (56.7% women), the mean chronological age was 65.1 years (Table 1). Cancer survivors were biologically older than non-cancer individuals, with greater PhenoAgeAccel in cancer survivors. Compared to matched non-cancer individuals, cancer survivors were more likely to be non-Hispanic White, alcohol drinkers, and smokers and have higher education, a higher number of comorbidities, and a higher total energy intake (all *p* < 0.05). The mean follow-up duration was 123.3 and 134.2 months for cancer survivors and matched non-cancer individuals, respectively. By the end of 2019, 2,526 participants in the study cohort (42.3%) died, including 698 cancer survivors (CVD-specific: 21.9%; cancer-specific: 30.9%) and 1,828 matched non-cancer individuals (CVD-specific: 29.8%; cancer-specific: 17.4%). The difference in cancer-specific deaths between cancer survivors and matched non-cancer individuals was statistically significant (*p* < 0.001), while the difference in CVD-specific deaths was marginally significant (*p* = 0.053).

**TABLE 1:** Distribution of characteristics based on cancer history status for adults from NHANES 1999–2010.

Characteristics	Total sample (n = 5,972)	Cancer history status		*p*-value
		Yes (n = 1,493)	No (n = 4,479)	
Chronological age, years (SE)	65.1 (15.2)	65.1 (15.2)	65.1 (15.2)	0.95
PhenoAgeAccel, years (SE)	0.0 (6.1)	0.8 (6.6)	−0.3 (6.0)	<0.001
Sex, n (%)	​	​	​	0.75
Male	2,591 (43.4%)	653 (43.7%)	1,938 (43.3%)	​
Female	3,381 (56.6%)	840 (56.3%)	2,541 (56.7%)	​
Race/ethnicity, n (%)	​	​	​	<0.001
Non-Hispanic white	3,615 (60.5%)	1,049 (70.3%)	2,566 (57.3%)	​
Non-Hispanic black	939 (15.7%)	217 (14.5%)	722 (16.1%)	​
Hispanic	1,240 (20.8%)	189 (12.7%)	1,051 (23.5%)	​
Other race^a^	178 (3.0%)	38 (2.5%)	140 (3.1%)	​
Educational level, n (%)	​	​	​	<0.001
Less than high school	1,956 (32.8%)	421 (28.2%)	1,535 (34.3%)	​
High school	1,469 (24.6%)	392 (26.3%)	1,077 (24.0%)	​
More than high school	2,547 (42.6%)	680 (45.5%)	1,867 (41.7%)	​
Alcohol drinking status, n (%)	​	​	​	0.003
Ever	4,956 (83.0%)	1,277 (85.5%)	3,679 (82.1%)	​
Never	1,016 (17.0%)	216 (14.5%)	800 (17.9%)	​
Smoking status, n (%)	​	​	​	<0.001
Current	873 (14.6%)	257 (17.2%)	616 (13.8%)	​
Former	2,182 (36.5%)	592 (39.7%)	1,590 (35.5%)	​
Never	2,917 (48.8%)	644 (43.1%)	2,273 (50.7%)	​
Physical activity level, n (%)	​	​	​	0.64
Inactive	3,240 (54.3%)	805 (53.9%)	2,435 (54.4%)	​
Moderate	2,196 (36.8%)	545 (36.5%)	1,651 (36.9%)	​
Active	536 (9.0%)	143 (9.6%)	393 (8.8%)	​
Body mass index, n (%)	​	​	​	0.84
<25	1,723 (28.9%)	439 (29.4%)	1,284 (28.7%)	​
25–30	2,181 (36.5%)	538 (36.0%)	1,643 (36.7%)	​
≥30	2,068 (34.6%)	516 (34.6%)	1,552 (34.7%)	​
Comorbidity index^c^, score (SD)	1.0 (1.1)	1.1 (1.2)	1.0 (1.1)	<0.001
Total energy intake	1,817.8 (809.0)	1,855.4 (795.9)	1,805.3 (813.0)	0.036
Duration of follow-up^b^, months (SD)	131.5 (56.2)	123.3 (56.7)	134.2 (55.8)	<0.001
Mortality status, n (%)	​	​	​	<0.001
Alive	3,446 (57.7%)	795 (53.2%)	2,651 (59.2%)	​
Deceased	2,526 (42.3%)	698 (46.8%)	1,828 (40.8%)	​
Leading cause of death, n (%)	​	​	​	​
Cardiovascular disease (CVD)	697 (27.6%)	153 (21.9%)	544 (29.8%)	0.053
Cancer	534 (21.1%)	216 (30.9%)	318 (17.4%)	<0.001

^a^ Other race: included non-Hispanic Asian and multiracial persons.

^b^ Duration of follow-up: from the NHANES interview date to 31 December 2019.

c The comorbidity index was constructed using seven self-reported conditions by a physician, namely, diabetes, coronary heart disease, cognitive heart failure, heart attack, stroke, hypertension, and liver disease.

**FIGURE 1:**
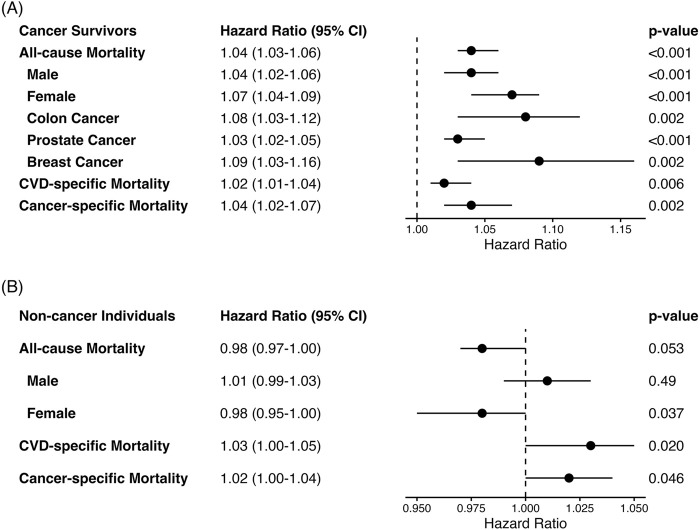
Forest plots of association between PhenoAgeAccel and risk of mortality based on the cancer history status ^*^ **(A)** Forest plots of HR and 95% CI in cancer survivors ^a, b^. **(B)** Forest plots of HR and 95% CI in individuals without cancer history ^a^. ^*^ Adjusted for survey weight, sex, race/ethnicity, educational level, alcohol drinking status, smoking status, body mass index (BMI), physical activity, comorbidity index, and total energy intake in analyses based on the cancer history status. ^a^ Stratified analysis by sex and adjusted for survey weight, race/ethnicity, educational level, alcohol drinking status, smoking status, BMI, physical activity, comorbidity index, and total energy intake. ^b^ For sample size consideration, analyses for the association between PhenoAgeAccel and all-cause mortality were limited among cancer survivors who had a history of colon, breast (female), or prostate (male) cancer diagnosis.

The associations between PhenoAge acceleration and mortality in cancer survivors and non-cancer individuals are shown in Figures 1A,B, respectively. In cancer survivors, greater PhenoAgeAccel was associated with an increased risk of all-cause (HR = 1.04, 95% CI: 1.03–1.06), CVD-specific (HR = 1.02, 95% CI: 1.04–1.04), and cancer-specific (HR = 1.04, 95% CI: 1.02–1.07) mortality (Figure 1A). The positive association between PhenoAgeAccel and all-cause mortality risk was stronger in female than in male cancer survivors and among breast cancer survivors than in prostate cancer survivors or colon cancer survivors. Similar patterns were observed among the matched non-cancer individuals (Figure 1B). However, the HR estimates were attenuated for all-cause and cancer-specific mortality (all-cause: HR = 0.98, 95% CI: 0.97–1.00; CVD-specific: HR = 1.03, 95% CI: 1.00–1.05; cancer-specific: HR = 1.02, 95% CI: 1.00–1.04), which may be due to the relatively short follow-up period for matched non-cancer individuals. The positive association between PhenoAgeAccel and all-cause mortality was limited in non-cancer male individuals but not in non-cancer female individuals.

The associations between cancer history and mortality were evaluated in the whole study cohort (Table 2). Compared to non-cancer individuals, cancer survivors had a significantly increased risk of all-cause (HR^TE^ = 1.14, 95% CI: 1.05–1.26) and cancer-specific mortality (HR^TE^ = 1.41, 95% CI: 1.15–1.82), while there was a marginal association between the cancer history status and CVD-specific mortality risk (HR^TE^ = 1.23, 95% CI: 0.99–1.50). In sensitivity analyses, results on the associations between PhenoAgeAccel and mortality risk among cancer survivors were not materially different; the associations between cancer history and higher risk of all-cause and cancer-specific mortality were also not materially different (data not shown).

**TABLE 2:** Association between the cancer history status and risk of mortality mediated through PhenoAgeAccel^a^.

Mortality	Indirect effect HR^IE^ (95% CI)	Direct effect HR^DE^ (95% CI)	Total effect HR^TE^ (95% CI)	% Mediated^b^
All-cause	1.02 (1.01, 1.03)	1.12 (1.03, 1.24)	1.14 (1.05, 1.26)	15.5
CVD-specific	1.03 (0.99, 1.08)	1.19 (0.96, 1.45)	1.23 (0.99, 1.50)	18.0
Cancer-specific	1.08 (1.02, 1.18)	1.31 (1.04, 1.72)	1.41 (1.15, 1.82)	24.1

^a^ Adjusted for race/ethnicity, sex, educational level, alcohol drinking status, smoking status, body mass index (BMI), physical activity, total energy intake, comorbidity index, and survey weight.

^b^ % Mediated was calculated by indirect effect/total effect, and therefore, it depends on both total and indirect effects.

We further conducted mediation analysis on the association between cancer history status, biological aging, and mortality risk in the whole study cohort (Table 2). Biological aging measured by PhenoAgeAccel mediated 15.5% of the association between cancer history and risk of all-cause mortality; the indirect association mediated through accelerated PhenoAge in the whole study cohort was 1.02 (95% CI: 1.01–1.03). A significant indirect association mediated through PhenoAge acceleration was observed for the positive association between cancer history and cancer-specific mortality (24.1%), with an HR^IE^ of 1.08 (95% CI: 1.02–1.18). There was no evidence for the mediation effect of PhenoAgeAccel on the association between cancer history status and CVC-specific mortality.

## Discussion

Utilizing data from six consecutive NHANES cycles, we found that, compared to individuals without a cancer history, cancer survivors had accelerated biological aging measured by PhenoAge; accelerated PhenoAge was associated with an increased risk of all-cause, cancer-specific, and CVD-specific mortality. These findings are consistent with previous reports suggesting positive associations between cancer history and acceleration of biological aging (Dugué et al., 2018; Beynon et al., 2022; Zhang et al., 2022; Wang et al., 2025). Cancer survivors may experience age acceleration because of direct and indirect impacts of cancer and its treatments (i.e., radiation, chemotherapy, and immunotherapy), which can cause cellular senescence, genomic instability, telomere attrition, epigenetic changes, or intercellular communication changes (Wang et al., 2021; Bhatia et al., 2022). However, the underlying mechanisms are yet to be elucidated.

With advances in detection and treatments, cancer survival has increased dramatically, with 67% of cancer survivors living ≥5 years and 18% living ≥20 years in the U.S. (American Cancer Society, 2019). However, cancer survivors continue to have a higher risk of mortality than individuals without a cancer history (Weaver et al., 2012; Lustberg et al., 2023), possibly through acceleration of biological aging caused by cancer and its treatments. Based on the mediation analyses, we found that PhenoAge mediated approximately 16% of the association between cancer history and all-cause mortality. Our analyses further showed that PhenoAge acceleration mediated 24.1% of the positive association between cancer history and risk of cancer-specific mortality, while a non-significant mediation effect of PhenoAgeAccel was observed for the association between cancer history and CVD-specific mortality. Consistent with previous studies (Zaorsky et al., 2017), this study showed that cancer was the leading cause of death, and CVD was the most common cause of non-cancer death among adults with a cancer history. The relatively small number of deaths from CVD may limit our ability to detect a weak association between cancer history and CVD-specific mortality and the possible mediating role of PhenoAge in this association. Moreover, we observed greater PhenoAge acceleration at the baseline among cancer survivors who died from cancer than those who died from CVD (data not shown), suggesting that cancer survivors who died from cancer might experience a greater biological aging process due to their diseases (i.e., stage and histological type) and treatment than those who died from CVD. Our findings also suggest that intervention strategies to ameliorate biological aging in cancer survivors may improve survivorship for this growing population. However, further studies, particularly large longitudinal studies, are needed to replicate our findings and elucidate the biological mechanisms underlying these relationships.

The strengths of our study include its population-based design with a representative sample of the U.S. population and detailed information on a wide range of potential confounding factors. We conducted sensitivity analyses by excluding participants with cancer diagnosis ≤5 years prior to entry into NHANES to reduce the potential for reverse causation as an explanation for the observed associations. However, our study may be limited by the use of self-reported cancer history data, which could introduce recall bias. The period for cancer diagnosis is long; both cancer history status and biomarkers were assessed cross-sectionally at baseline, suggesting that their temporal sequences may not be clear. We cannot discount the possible long-term influence of pre-diagnosis biological age acceleration on mortality, although the results remained persistent in sensitivity analysis. In addition, despite adjustment for many potential confounding factors in the analyses, unmeasured factors such as tumor characteristics (e.g., stage and histological type) and specific cancer treatments may still lead to residual confounding. Finally, there is no gold standard for measuring biological aging currently. PhenoAge was used to measure biological aging in the current analysis, whereas several clinic biomarker-based approaches have been developed (Belsky et al., 2018), and different measures may not reflect the same process of biological aging. Replication of our results in other populations with PhenoAge and other biomarker-based measures of biological age is warranted to confirm the mediating effect of biological aging in the associations between cancer history and mortality risk.

In summary, the current study found that cancer survivors experience greater PhenoAge acceleration than individuals without a cancer history; biological aging, measured by clinical-marker-based PhenoAge, mediated the association between cancer history and mortality, particularly cancer-specific mortality. Further prospective studies with larger sample sizes of cancer survivors are warranted to confirm these findings. Studies exploring intervention strategies to slow age acceleration in cancer survivors are needed.

## Data Availability

Data used in this study are publicly available and can be accessed through NHANES website: https://wwwn.cdc.gov/nchs/nhanes/default.aspx.
